# Newborn genome-wide DNA methylation in association with pregnancy anxiety reveals a potential role for *GABBR1*

**DOI:** 10.1186/s13148-017-0408-5

**Published:** 2017-10-03

**Authors:** Elise Beau Vangeel, Ehsan Pishva, Titia Hompes, Daniel van den Hove, Diether Lambrechts, Karel Allegaert, Kathleen Freson, Benedetta Izzi, Stephan Claes

**Affiliations:** 1 Department of Neurosciences, Genetic Research About Stress and Psychiatry (GRASP), KU Leuven, Leuven, Belgium; 2 Department of Cardiovascular Sciences, Center for Molecular and Vascular Biology (CMVB), KU Leuven, Leuven, Belgium; 3 Department of Psychiatry and Neuropsychology, School for Mental Health and Neuroscience, Maastricht University Medical Centre, Maastricht, The Netherlands; 4 University of Exeter Medical School, University of Exeter, Exeter, UK; 5 University Psychiatric Center, Leuven, Belgium; 6 Department of Psychiatry, Psychosomatics and Psychotherapy, Laboratory of Translational Neuroscience, University of Wuerzburg, Wuerzburg, Germany; 7 Department of Oncology, Laboratory of Translational Genetics, KU Leuven, Leuven, Belgium; 8 Vesalius Research Center (VRC), VIB, Leuven, Belgium; 9 Department of Development and Regeneration, KU Leuven, Leuven, Belgium; 10 Intensive Care and Department of Pediatric Surgery, Erasmus MC—Sophia’s Children’s Hospital, Rotterdam, The Netherlands; 11 Department of Epidemiology and Prevention, IRCCS Instituto Neurologico Mediterraneo Neuromed, Pozzilli, Italy

**Keywords:** DNA methylation, Pregnancy anxiety, *GABBR1*, Prenatal stress, Gender differences, HPA axis

## Abstract

**Background:**

There is increasing evidence for the role of prenatal stress in shaping offspring DNA methylation and disease susceptibility. In the current study, we aimed to identify genes and pathways associated with pregnancy anxiety using a genome-wide DNA methylation approach.

**Methods:**

We selected 22 versus 23 newborns from our Prenatal Early Life Stress (PELS) cohort, exposed to the lowest or highest degree of maternal pregnancy anxiety, respectively. Cord blood genome-wide DNA methylation was assayed using the HumanMethylation450 BeadChip (HM450, *n* = 45) and candidate gene methylation using EpiTYPER (*n* = 80). Cortisol levels were measured at 2, 4, and 12 months of age to test infant stress system (re)activity.

**Results:**

Data showed ten differentially methylated regions (DMR) when comparing newborns exposed to low versus high pregnancy anxiety scores. We validated a top DMR in the GABA-B receptor subunit 1 gene (*GABBR1*) revealing the association with pregnancy anxiety particularly in male newborns (most significant CpG Pearson *R* = 0.517, *p* = 0.002; average methylation Pearson *R* = 0.332, *p* = 0.039). Cord blood *GABBR1* methylation was associated with infant cortisol levels in response to a routine vaccination at 4 months old.

**Conclusions:**

In conclusion, our results show that pregnancy anxiety is associated with differential DNA methylation patterns in newborns and that our candidate gene *GABBR1* is associated with infant hypothalamic-pituitary-adrenal axis response to a stressor. Our findings reveal a potential role for *GABBR1* methylation in association with stress and provide grounds for further research.

**Electronic supplementary material:**

The online version of this article (10.1186/s13148-017-0408-5) contains supplementary material, which is available to authorized users.

## Background

Prenatal stress exposure can adversely influence infant development and alter susceptibility to obesity, metabolic disorders, and mental health outcomes [1]. Although the exact mechanisms of such links between the prenatal environment and postnatal (e.g., behavioral) phenotype are still unknown, many recent studies found evidence of a potential role for DNA methylation. Different forms and severity of maternal psychosocial stress were found to have an influence on fetal DNA methylation patterns [2]. Epigenetic studies investigating maternal well-being during pregnancy have largely made use of candidate gene approaches. For instance, gene-specific DNA methylation changes associated with prenatal stress were found in the glucocorticoid receptor gene (*NR3C1*) [3]. More specifically, studies showed associations of infant *NR3C1* DNA methylation with maternal depressive symptoms [4–6], exposure to inter-partner violence [7], and war stress [8, 9]. A link of *NR3C1* DNA methylation and altered infant stress reactivity of the hypothalamic-pituitary-adrenal (HPA) axis was found in the study of Oberlander et al. [4]. DNA methylation of other candidate genes with a role in HPA axis [6, 9] or immune system function [10] was associated with prenatal stress as well.

In addition to these candidate gene approaches, some epigenome-wide association studies (EWAS) have examined the potential role of prenatal stress. Genome-wide DNA methylation changes have been found in newborns from women with non-medicated depression or anxiety during pregnancy [11], while another study shows an association between the methyltransferase pathway and prenatal stress in two population-based cohorts [12]. Contrasting maternal perceived stress and objective measurements of hardship, Cao-Lei and colleagues found that only the latter was associated with DNA methylation at several CpG sites of children in the Project Ice Storm study [2]. Furthermore, a recent study by Serpeloni et al. described genome-wide DNA methylation changes associated with grandmaternal exposure to violence during pregnancy [13].

In previous publications examining our specific birth cohort, we have shown that pregnancy anxiety appeared to be an important factor associated with DNA methylation changes of the *NR3C1* gene [14] and two imprinted genes involved in fetal development, i.e., insulin-like growth factor 2 (*IGF2*) and guanine nucleotide binding protein, alpha stimulating (*GNAS*) in cord blood [15]. These studies provide evidence that not only extreme forms of prenatal stress such as exposure to war or natural disasters [2, 8] but also maternal anxiety in a general healthy population, can alter DNA methylation in the offspring.

Following up on our candidate gene studies, we here performed an EWAS to identify the DNA methylation changes in newborns associated with maternal pregnancy-related anxiety. This hypothesis-generating approach provides the opportunity to identify novel candidate genes but also to study interactions using a biological pathway analysis. The magnitude of one of our EWAS discovery findings was subsequently investigated in an extended sample set using a different DNA methylation quantification technique, the Sequenom EpiTYPER platform. Finally, we examined the association of DNA methylation at our candidate region with infant HPA axis function.

## Methods

### Study population

Between October 2009 and December 2010, samples from 170 highly educated pregnant women at 6 to 12 weeks of gestation were recruited within the Prenatal Early Life Stress (PELS) study in the University Hospitals Leuven, Belgium. A detailed description of data and sample collection can be found elsewhere [14].

Briefly, each trimester, the pregnant women completed several questionnaires regarding mental health and general well-being. From 80 of these 170 participating mothers, we were able to collect a cord blood sample at birth, of which DNA was subsequently isolated. Of these 80 samples, 45 DNA samples were selected for genome-wide DNA methylation analysis based on maternal psychological measurements, while all 80 DNA samples were subsequently used for the candidate gene confirmation study. A schematic overview of the study design is represented in Additional file 1: Figure S1.

### Psychological measurements

At each trimester of pregnancy, future mothers were asked to complete questionnaires assessing depression, anxiety, and mother-fetus relationship. The revised pregnancy-related anxiety questionnaire (PRAQ) was used to assess worries and anxiety specifically regarding the participating mother’s pregnancy [16]. The validated revised version, which we used for the current study, contains three subscales: (1) fear of integrity of the fetus, (2) concerns about one’s own appearance, and (3) fear of the delivery itself [17]. We previously reported a high internal reliability for the total scale (Cronbach’s *α* ≥ 0.95) and good internal reliability for the subscales (Cronbach’s *α* ≥ 0.73) of the revised questionnaire [14]. To select the samples for EWAS, we focused on the fear of integrity subscale of PRAQ, which appeared as most associated with DNA methylation in our candidate gene studies [14, 15]. This subscale provides a score ranging from zero to seven and measures maternal anxiety regarding the health of the unborn infant and fear of integrity of the newborn.

### Child HPA axis measurements

In order to measure infant HPA axis stress response as well as baseline activity, cortisol was measured in response to a vaccination at 2 and 4 months old and during the day at 12 months old. In both vaccination studies and the day profile study, saliva samples for cortisol measurements were taken using BD Visitec eye sponges (Becton, Dickson and Company, Waltham, USA). To determine cortisol levels in saliva a High Sensitivity Salivary Cortisol Enzyme Immunoassay Kit (ELISA kit, Salimetrics, Europe) was used. This assay captures the full range of salivary cortisol levels (0.003 to 3.0 μg/dL) while using only 25 μL of saliva per test and it was designed by Salimetrics to be resilient to the effects of interference caused by collection techniques that affect pH.

Cortisol was measured in response to a physical stressor at 2 and 4 months of age. All visits were scheduled in the afternoon to control for circadian rhythm. Mothers were asked not to feed their infants from 1 hour before the test until after the test since this might compromise assay performance by lowering sample pH and influencing bacterial growth. The routine vaccination consisted of two intramuscular injections: one injection for *Pneumococcus* and one combined injection for diphtheria, whooping cough, tetanus, poliomyelitis, and *Haemophilus* influenza type B. Saliva for cortisol measurements was collected at arrival to the examination room and at 15, 30, 45, and 60 min following the injection. The time between arrival and vaccination included the time when the parent undressed the infant, when both waited for the doctor to arrive, and when the doctor was taking the health history and the physical examination. The use of this vaccination stressor has a number of advantages: (1) it is routinely performed on all infants and therefore is convenient; (2) it is standardized, and so equal treatment to all infants can be assumed (especially if all the data are collected at the same facility); (3) the onset of the application of the aversive stimulation can be precisely determined; and (4) the inoculation is aversive enough to evoke behavioral distress [18]. The maximum value following vaccination was noted and the area under the curve with respect to increase (AUCi), initial response (value at 15 min minus baseline), and recovery slope (value at 60 min minus 15 min) were calculated. Data for several of these variables were skewed (Shapiro-Wilk *p* value < 0.05) and were logit transformed to correct skewness. If measurements for one-time point were missing, AUCi, initial response and recovery slope were not calculated for this individual.

When infants were 12 months of age, mothers were instructed to collect saliva samples of their infant at awakening, 30 min, 4, and 12 h after awakening. Samples were sent back to our lab in a prepaid, addressed envelope, and processed to measure cortisol levels. The cortisol awakening response (CAR) was calculated and a repeated *t* test was carried out to verify a significant cortisol increase between awakening and 30 min.

### Illumina Infinium HumanMethylation450 BeadChip Assay

Cord blood DNA samples were processed by the Barts and the London Genome Centre (London, UK), where bisulfite treatment was carried out using the Zymo EZ DNA methylation kit (Zymo Research) prior to running the Infinium HumanMethylation450 BeadChip (HM450) arrays (Illumina Inc., California, USA). In total, 48 samples were randomized over four chips based on PRAQ score, maternal smoking, maternal alcohol use, gender of the newborn, parity and gravidity, gestational age at birth, and maternal age. One sample was replicated on each chip to account for batch effects [19], resulting in a sample size of 45 unique samples analyzed on the HM450 array.

### Data processing and quality control

Raw data generated by the iScan Illumina array were imported using GenomeStudio software (Illumina, Inc.) and the subsequent quality control and normalization were implemented using the wateRmelon package in R (available from the Bioconductor repository http://www.bioconductor.org) [20]. Data clean-up included removal of samples and CpG probes with insufficient data quality. In more detail, samples were removed if more than 5% of its sites had a poor detection *p* value (> 0.01), and probes with a detection *p* value of more than 0.01 in more than 1% of samples or a bead count of less than 3 in more than 5% of samples were removed from the analysis. Furthermore, cross-hybridizing probes, probes containing a common single nucleotide polymorphism (SNP) in the sequence or within 10 bp of the sequence, and probes on the X and Y chromosomes were removed [21]. The final analysis included 414,733 probes, and all samples passed the stringent quality control. A schematic overview of the analysis from data preprocessing until validation can be found in the Supplementary information (Additional file 1: Figure S1).

### Genome-wide DNA methylation analysis

Differentially methylated regions (DMRs) were first explored using the R package *DMRcate* [22]. The *DMRcate* statistical model was corrected for gender, gestational age of the newborn at birth, batch (chip Sentrix ID), and cell type compositions as calculated with the Houseman algorithm [23]. Using the top 500 uniquely annotated DMRs from *DMRcate*, we performed a pathway analysis. Enrichment for Gene Ontology (GO) classes was conducted using the over-representation analysis (ORA) method in ErmineJ with a minimum gene set of 5, and a maximum of 100 [24].

### DMR verification and validation using Sequenom EpiTYPER

In order to verify the top DMRs from the *DMRcate* analysis, we used a second region-specific method: the Python module *comb-p* [25]. *Comb-p* employs probe locations and *p* values from the differentially methylated CpG probe (DMP) analysis as input to identify differentially methylated regions. We, therefore, first calculated DMPs using a linear regression model with pregnancy anxiety as a categorical measurement (high versus low) corrected for gender, gestational age of the newborn, batch (chip Sentrix ID) effects, and cell type compositions, similar to the *DMRcate* model. A list of DMPs was further used for *comb-p* DMR analysis. Both *comb-p* and *DMRcate* have distinct underlying statistical methods to identify DMRs. *DMRcate* runs a model using a kernel smoothing function on logit-transformed beta values (“M values”), independently from the single CpG site significance. *Comb-p*, on the contrary, identifies DMRs based on the output of a probe-by-probe analysis, i.e., using the DMP analysis *p* values and locations of each CpG probe. Both methods calculate DMRs only accounting for their location in the genome, i.e., non-annotated. We combined their results to rank DMRs and further use this ranking to select candidate genes. To statistically test the overlap of unique, protein-coding genes between *DMRcate* and *comb-p* lists, the R package GeneOverlap (Bioconductor) was used [26].

Based on the HM450-identified DMR, an amplicon of 446 base pairs was designed using EpiDesigner. A schematic overview and the genomic sequence of the amplicon located at chr6:29594930–29595375 (hg19; forward primer: GGTTAGGGGGTTAGGTTTGTTAGTT; reverse primer: ACTCCCTCAAAAAATCAATATCTCC) can be found in Additional file 1: Figure S2. In silico analysis, using the MassArray package in Bioconductor [27] preceded laboratory experiments in order to assess (un)successful CpG units in advance [28]. EpiTYPER DNA methylation analysis was performed for a total of 80 cord blood samples (including 45 samples used for the HM450 analysis and 35 additional samples) of the PELS cohort as described [15]. In short, cord blood DNA (1 μg) was bisulfite treated using the MethylDetector kit with long cycling protocol (Active Motif, Carlsbad, CA, USA) [29]. Subsequently, amplicons were amplified in triplicate for each sample, followed by T-cleavage reactions and detection by mass spectrometry using the Sequenom MassARRAY (San Diego, CA, USA) protocol. The signal was subsequently translated by the EpiTYPER software resulting in DNA methylation percentages for each CpG unit. Values were excluded if the standard deviation between triplicates was more than 10%, and CpG units were excluded when having a success rate of less than 70%. A summary of CpG sites analyzed by EpiTYPER and their corresponding HM450 CpG probe identifiers is shown in the Supplementary data (Additional file 2). For this part of the study, all 80 samples of our PELS cohort were used and, therefore, PRAQ could be analyzed as a continuous variable. The association of DNA methylation at each CpG unit with PRAQ was analyzed using Pearson’s correlation tests. SPSS Statistics, version 23 (IBM Corp., Armonk, NY, USA) was used for these statistical analyses.

## Results

### Demographic data of the study cohort selected for EWAS

Cord blood DNA samples for the genome-wide methylation analysis were selected from the 80 mother-infant dyads of our PELS cohort. We aimed for the upper and lower quartiles of PRAQ-integrity scores and included 23 mothers with the highest and 22 mothers with the lowest scores.

All participating mothers were European, with a mean maternal age of 30.6 years and an average length of gestation of 277.2 days. An overview of selected demographic parameters and comparison between the high and low pregnancy anxiety group can be found in Table 1.

**Table 1:** Study cohort for the genome-wide DNA methylation analysis with low versus high maternal pregnancy anxiety (PRAQ): fear of integrity scores

	Low pregnancy anxiety	High pregnancy anxiety		
	*n* = 22	*n* = 23	Test statistic	*p* value
Pregnancy anxiety (PRAQ score)	2.1 (0.4)	5.2 (0.7)	*t* = − 18.906	*p* < 0.0001
Maternal age (years (SD))	30.0 (2.5)	31.1 (4.0)	*t* = − 1.115	0.27
Paternal age (years (SD))	32.0 (4.0)	32.5 (4.6)	*t* = − 0.397	0.69
Single parenthood, *n* (%)	0	1 (4.3%)	Fisher’s exact	1
Method of birth, *(n* cesarean section (%))	2 (9.1%)	8 (34.8%)	Fisher’s exact	0.07
Maternal BMI before pregnancy (kg/m^2^)	23.3 (3.5)	23.4 (4.3)	*t* = − 0.478	0.64
Maternal smoking, *n* (%)	2 (9.1)	0	Fisher’s exact	0.23
Maternal alcohol use, *n* (%)	2 (9.1)	4 (17.4)	Fisher’s exact	0.67
Gender of the baby, female *n* (%)	11 (50)	10 (43.5)	Chi squared = 0.192	0.77
Gestational age (days (SD))	277.3 (7.2)	277.1 (10.2)	*t* = 0.071	0.94
Birth weight (g (SD))	3544.1 (426.1)	3293.5 (447.2)	*t* = 1.923	0.06

### Global DNA methylation is not influenced by pregnancy anxiety

Global DNA methylation difference between the low and the high prenatal anxiety group was assessed. We compared DNA methylation of all long interspersed nuclear element (LINE) repeats by analyzing preprocessed and normalized beta values at CpG probes in both LINE-1 and LINE-2 repeats extracted from the HM450 data as described previously [30]. This analysis involved 15,612 CpG probes at LINE repeats localized throughout the genome. The average DNA methylation percentage for these repetitive elements was 68.58 versus 68.61% for the high and low prenatal anxiety group, respectively.

### Differentially methylated regions associated with pregnancy anxiety

Because a region of nearby CpG sites that are differentially methylated often provides stronger evidence for effective transcriptional regulation, we investigated the presence of DMRs using *DMRcate*. This method starts from preprocessed, normalized methylation values to analyze the HM450 data. *DMRcate* identified 901 DMRs with 3 or more probes that were differentially methylated between newborns exposed to high versus low pregnancy anxiety (top 50 DMRs presented in Additional file 3).

Epigenome-wide pathway analysis was performed in order to determine specific pathways, which are over-represented. For this purpose, we used ErmineJ on the top 500 uniquely annotated DMRs from *DMRcate*, enabling us to additionally assess multifunctionality of the resulting GO terms. The top enriched pathways are shown in Additional file 4, showing medium to high multifunctionality scores. Three of the top five GO terms relate to brain development, i.e., “pallium development” (*p* = 0.001), “hippocampus development” (*p* = 0.004), and “telencephalon development” (*p* = 0.004). Other top GO terms seem to be related to general cellular function and tight junctions with multifunctionality scores ranging from 0.002 to 0.932 (Additional file 4).

### *GABBR1* DNA methylation is positively associated with pregnancy anxiety in male newborns

We aimed at identifying a candidate gene to be further investigated in a larger sample set that includes the full PELS cohort (*n* = 80). For this purpose, we wanted to strengthen the DMR identification analysis by applying to our genome-wide data a second statistical method to identify DMRs, namely *comb-p*. The latter, in contrast to *DMRcate*, identifies DMRs based on the output of a probe-by-probe analysis, using the location and *p* values of DMPs. To this end, we first tested each CpG probe for differential methylation between neonates exposed to high or low pregnancy anxiety, while correcting for batch effects, gestational age, gender of the newborn, and cord blood cell type estimates (Additional file 5). In total, 17,180 CpG sites were differentially methylated with an unadjusted *p* value of < 0.05 (referred to as “DMP”) between the low and high pregnancy anxiety group. However, following the false discovery rate (FDR) correction for multiple testing none of the individual probes remained significant.

**Fig. 1:**
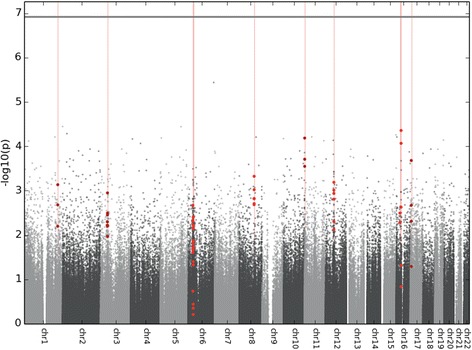
Manhattan plot showing the −log10 *p* values for all CpG probes and their genomic locations included in the genome-wide methylation analysis (*n* = 414,733). The false discovery rate *p* value threshold is represented by a horizontal line. Vertical lines and overlaid bold dots correspond to differentially methylated regions (DMRs) identified by *comb-p*, as represented in Table 2 [25]

Subsequently, the *comb-p* tool was applied, returning ten DMRs (Table 2). Individual probes within the DMRs identified by *comb-p* are highlighted in a Manhattan plot (Fig. 1). The top ten DMRs according to the *comb-p* method are presented in Table 2 with indication of the rank order of the equivalent genomic region in the *DMRcate* results. There was a significant overlap between genes annotated to the *DMRcate* and *comb-p* lists, as assessed by GeneOverlap (*p* = 3.4E-7). The combined ranking of both *comb-p* and *DMRcate* DMR analyses identified homeobox C4 (*HOXC4*), palmitoyl-protein thioesterase 2 (*PPT2*) and GABA-B receptor subunit 1 gene (*GABBR1*) as top three DMRs of interest with a maximum beta fold change (FC) of 0.044 (minimum *comb-p* *p* = 1.8E-06, *DMRcate* *p* = 5.36E-14), 0.050 (minimum *comb-p* *p* = 4.5E-05, *DMRcate* *p* = 5.43E-18), and 0.053 (minimum *comb-p* *p* = 0.00015, *DMRcate* *p* = 1.08E-08), respectively. Contrary to *HOXC4* and *PPT2*, *GABBR1* DMR is located within a CpG island near active regulatory elements (Additional file 1: Figure S2) and was therefore selected for our validation study with Sequenom EpiTYPER. DNA methylation levels for each HM450 CpG probe in the *GABBR1* DMR identified by *DMRcate*, including percent methylation difference between both groups (range 0.24 to 4) and effect sizes (range 0.170 to 0.825) can be found in Additional file 6.

**Table 2:** *Comb-p* determined differentially methylated regions (DMR)

Rank *comb-p*	Rank *DMRcate*	RefGene name	Location (GRCh37/hg19 assembly)	Minimum *p* value	Number of probes	Stouffer-Liptak-Kechris *p* value	Šidák corrected	CpG island
1	624	TP53INP1	chr8:95962083–95962464	0	6	0	0	yes
2	NA	PRSS50	chr3:46759334–46759699	0	8	0	0	yes
3	3	*HOXC4*	chr12:54446278–54446577	0	6	0	0	no
4	169	PKP3	chr11:396685–397078	0	3	0	0	yes
5	NA	ZNF764	chr16:30572738–30573014	0	5	0	0	yes
6	2	*PPT2*	chr6:32120954–32121421	0	14	0	0	no
7	21	*GABBR1*	chr6:29595001–29595316	0	7	2.70E-07	0.07	yes
8	358	PITPNM3	chr17:6358362–6358600	0	4	2.88E-07	0.1	yes
9	NA	ESRRG	chr1:217306589–-217306764	0.01	3	1.01E-06	0.39	no
10	NA	HS3ST2	chr16:22959593–22959820	0	2	0	1	yes

Both uncorrected (Stouffer-Liptak-Kechris) *p* values and *p* values corrected for multiple testing (Sidak) are shown, as well as the rank order of the DMR obtained using the *DMRcate* method

**Fig. 2:**
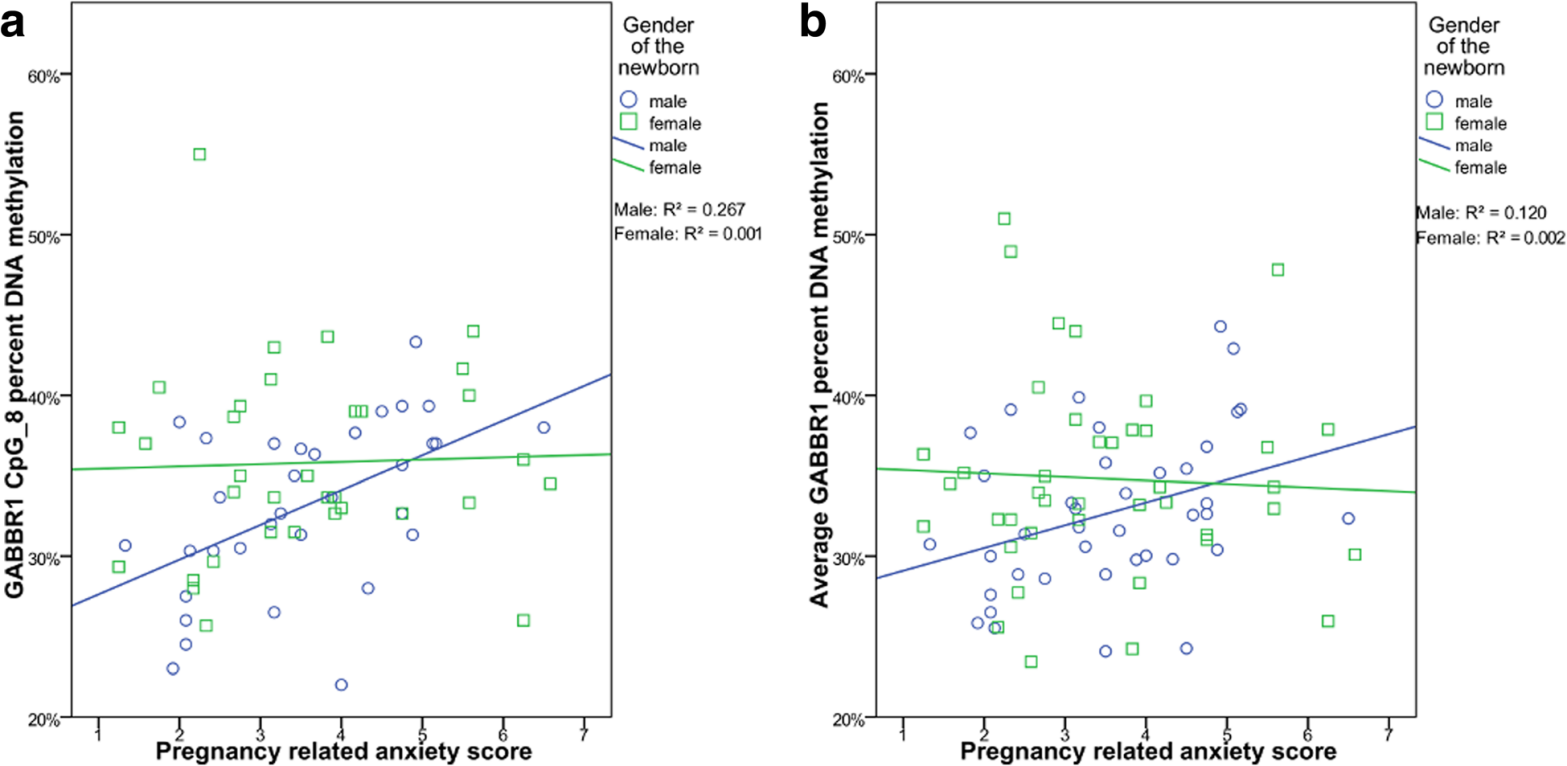
Correlations for male (blue; *n* = 39) and female (green; *n* = 41) newborns between pregnancy anxiety (PRAQ) integrity scores and GABA-B receptor subunit 1 gene (*GABBR1*) DNA methylation at **a** the most significant CpG site (CpG_8) and **b** on average across the DMR

An overview of the *GABBR1* gene and the region analyzed by Sequenom EpiTYPER platform can be found in Additional file 1: Figure S2. A summary of CpG units analyzed or excluded for technical reasons is shown in Additional file 2 and correlations between EpiTYPER and HM450 probes are shown in Additional file 7. The 17 CpG sites present in the *GABBR1* DMR corresponded to 14 CpG units, of which 4 could not be analyzed due to technical reasons inherent to the MassArray and EpiTYPER methodology (e.g., low mass, fragment overlap) and one had a low success rate (CpG_4). Correlations between pregnancy anxiety and DNA methylation of the *GABBR1* DMR were not significant in the total sample set (mean *GABBR1* DMR methylation: Pearson *R* = 0.100, *p* = 0.379). However, as DNA methylation at several *GABBR1* CpG units was associated with the pregnancy anxiety x gender interaction term (CpG_8 *p* = 0.045, CpG_9.10 *p* = 0.014, CpG_14.15 *p* = 0.029, and a trending effect at CpG_3 *p* = 0.06), data were stratified by gender. When analyzing the EpiTYPER data for this *GABBR1* separately for male and female newborns, we observed a positive correlation between fetal *GABBR1* DNA methylation and pregnancy anxiety in male newborns on average across the amplicon (Pearson *R* = 0.332, *p* = 0.039), at CpG_6, CpG_8, CpG_9.10, CpG_14.15, and a trend was found (*p* < 0.1) at CpG_1.2, CpG_16, and CpG_17 (Table 3). Only single CpG units CpG_8 (Pearson *R* = 0.517, *p* = 0.002) and CpG_14.15 (Pearson *R* = 0.462, *p* = 0.003) remained significant following Bonferroni correction for multiple tests (at *α* = 0.0056) (Fig. 2).

**Table 3:** Pearson correlations of pregnancy anxiety (PRAQ) integrity scores with cord blood *GABBR1* DNA methylation (average and separate CpG units) generated by EpiTYPER, for boys (*n* = 39) and girls (*n* = 41) separately

		*GABBR1* DNA methylation									
		CpG_1.2	CpG_3	CpG_6	CpG_8	CpG_9.10	CpG_11	CpG_14.15	CpG_16	CpG_17	Average *GABBR1*
Boys (*n* = 39)	Pearson *R*	0.302	0.184	0.37	0.517^a^	0.37	0.156	0.462^a^	0.295	0.299	0.33
	*p* value	0.061	0.306	0.043	0.002	0.023	0.395	0.003	0.095	0.086	0.039
Girls (*n* = 41)	Pearson *R*	− 0.029	− 0.288	0.032	0.035	− 0.192	− 0.128	− 0.040	− 0.078	− 0.106	− 0.049
	*p* value	0.860	0.093	0.858	0.844	0.278	0.451	0.824	0.655	0.531	0.761

^a^Significant after correction for tests at nine CpG sites (*α* = 0.0056)

### *GABBR1* DNA methylation significantly correlates with HPA axis function

Using EpiTYPER data (*n* = 80), we aimed to assess whether the DNA methylation changes in the *GABBR1* DMR are also functionally relevant. For this, we examined how cord blood *GABBR1* DNA methylation relates to cortisol levels during a stress response at 2 and 4 months and to the cortisol awakening response at 12 months of age. Information on the raw data of these cortisol measurements can be found in Additional file 1: Figure S3. At 2 and 4 months, infants were exposed to a physical stressor, more specifically a routine vaccination injection. Each time, five saliva samples were taken to measure the cortisol levels and calculate the elicited stress response. A paired *t* test confirmed a significant cortisol increase between T0 and 15 min at 2 months (*p* = 0.002) and 4 months (*p* < 0.001). At 2 months, one CpG unit was associated with the initial cortisol response to the stressor. At 4 months, several CpG units appeared to be associated with the AUCi, initial response and recovery slope (Table 4). However, following multiple testing corrections for 27 tests at each study moment (*α* = 0.0019), only the following associations remained. At 4 months, DNA methylation at CpG_1.2 was significantly associated with the initial response (Pearson *R* = 0.392, *p* = 0.001). This same CpG unit was significantly associated with the recovery slope at 4 months (Pearson *R* = 0.400, *p* = 0.001; Table 4).

**Table 4:** Pearson correlations of cord blood *GABBR1* DNA methylation (average and separate CpG units) with infant HPA axis cortisol measurements in response to routine vaccination at 2 and 4 months of age

			*GABBR1* DNA methylation									
Child HPA axis stress response			CpG_1.2	CpG_3	CpG_6	CpG_8	CpG_9.10	CpG_11	CpG_14.15	CpG_16	CpG_17	Average *GABBR1*
2 M (*n* = 20)	AUCi	Pearson *R*	0.372	− 0.476	0.029	− 0.452	0.109	0.062	0.375	0.238	0.218	− 0.152
		*p* value	0.141	0.053	0.924	0.091	0.678	0.826	0.153	0.375	0.418	0.547
	Initial response	Pearson *R*	0.141	− 0.47	0.092	− 0.270	0.120	− 0.004	0.448	0.125	0.172	− 0.098
		*p* value	0.565	0.043	0.745	0.295	0.624	0.989	0.062	0.621	0.494	0.680
	Recovery slope	Pearson *R*	0.141	− 0.283	0.063	− 0.162	0.118	− 0.103	0.342	− 0.012	0.083	− 0.086
		*p* value	0.576	0.255	0.830	0.550	0.641	0.716	0.179	0.964	0.751	0.727
4 M (*n* = 67)	AUCi	Pearson *R*	0.260	0.239	0.241	0.200	0.084	0.096	0.080	0.186	0.082	0.168
		*p* value	0.041	0.079	0.091	0.150	0.538	0.487	0.553	0.177	0.547	0.189
	Initial response	Pearson *R*	0.392^a^	0.215	0.35	0.3	0.166	0.184	0.077	0.160	0.136	0.202
		*p* value	0.001	0.102	0.009	0.023	0.210	0.166	0.556	0.230	0.301	0.100
	Recovery slope	Pearson *R*	0.400^a^	0.219	0.38	0.35	0.209	0.27	0.130	0.192	0.182	0.225
		*p* value	0.001	0.102	0.005	0.010	0.115	0.047	0.327	0.159	0.173	0.071

^a^Significant at the *α* level of 0.0019, corrected for 27 tests (3 cortisol variables and 9 CpG sites)

At 12 months, salivary cortisol levels of infants were measured to calculate cortisol awakening responses. However, performing a paired *t* test indicated that there was no significant increase in cortisol between awakening and 30 min (mean difference = − 0.009, *p* = 0.647). This is also apparent from Additional file 5: Figure S3B and S3C. Since there was no significant awakening response found, association analyses with *GABBR1* DNA methylation levels were not carried out.

## Discussion

In our PELS cohort, we found that prenatal exposure to maternal pregnancy-related anxiety is associated with region-specific DNA methylation changes across the genome. Previously, we have shown that pregnancy anxiety is linked to fetal DNA methylation in the glucocorticoid receptor gene (*NR3C1*), which is a key player in the hypothalamic-pituitary-adrenal axis, and to DNA methylation of imprinted genes *IGF2* and *GNASXL* important in fetal growth and development [14, 15]. This led us to hypothesize that PRAQ measurements, more specifically the “fear of integrity” subscale, could indeed have more widespread associations with fetal DNA methylation.

DNA methylation of repetitive sequences such as LINE elements have been associated with growth trajectories and birthweight [31]. However, in our study, global fetal DNA methylation was not different in the high versus low prenatal anxiety group and these groups did not cluster together.

Pathway and GO analysis using ErmineJ [24] results of the current study should be interpreted carefully, taking into account the multifunctionality scores of each GO term. High multifunctionality scores indicate that many genes involved have several different biological functions, complicating interpretation of these results. Our findings showed that the top-ranked DMRs annotated to genes identified in our study were enriched in pathways related to brain development, general cellular functions, and tight junctions.

We based the selection of the *GABBR1* gene for further validation on the merged analysis from both *DMRcate* and *comb-p*, two methods that use different strategies to identify DMRs using HM450 data. Although absolute DNA methylation differences appeared small, Cohen’s d effect sizes in the region were medium to large. Additionally, small absolute differences have previously been found to be relevant and correlated to biologically meaningful measurements [32, 33]. Interestingly, the EpiTYPER validation study of the *GABBR1* DMR on the full PELS cohort confirmed a gender-specific association of pregnancy anxiety with *GABBR1* DNA methylation. More specifically, in male neonates, CpG_8 and CpG_14.15 were significantly associated with pregnancy anxiety following multiple testing corrections, with a large effect size (Pearson correlation coefficients *R* = 0.517 and 0.462, respectively). None of the *GABBR1* DMR CpG units were significantly associated with pregnancy anxiety in female newborns. A possible explanation for the gender differences might be that the exposure to an adverse environment is processed in a different way by both genders. Already during fetal development differences in vulnerability to a range of diseases, including psychiatric disorders, arise between males and females [34] while susceptibility as well as presentation and therapeutic outcomes appear to be gender-specific for certain psychiatric disorders later in life [35]. Evidence from rodent and human research also indicates that the influence of prenatal environment on neurodevelopment is modulated by sex [36, 37]. Finally, gender-specific DNA methylation trajectories were found during fetal neurodevelopment [38], suggesting that gender is an important factor to take into account in analyses similar to the current study.

The identified candidate gene *GABBR1* encodes a G-protein coupled receptor subunit GABA-B1, which heterodimerizes with the GABA-B2 subunit to form the GABA-B receptor. Gamma-aminobutyric acid (GABA) can reduce neuronal excitability in the central nervous system by binding these GABA-B or the GABA-A receptors. GABA signaling has a crucial, yet complex, role in the neuroendocrine adaptation and neuronal plasticity in response to stress, mainly in the paraventricular nucleus of the hypothalamus [39]. GABAergic neurotransmission has been implicated in many psychiatric disorders, including anxiety, depression, and schizophrenia [40]. Compelling recent research reveals *GABBR1* methylation changes in HM450 data of a high-risk population of youths exposed to childhood maltreatment and further suggests that the identified markers may provide evidence for a molecular link between early life stress and mental health [41]. DNA methylation of the *GABBR1* gene has further been implicated in obsessive-compulsive disorder (OCD) [42] as well as schizophrenia [43]. In female OCD patients, *GABBR1* DNA methylation was found to be different from healthy controls and associated with symptom severity at baseline, effect of treatment, and responder status. In rats, *NR3C1* DNA methylation changes have been found in the hippocampal tissue of rat pups exposed to poor maternal care, which eventually displayed poor maternal care themselves and were more anxious in adult life. Furthermore, maternal care and early life stress in rats were linked to the GABAergic synapse and altered GABA-A receptor expression [44]. In the current study, we identified *GABBR1* DNA methylation as affected by prenatal anxiety exposure in a birth cohort. Interestingly, using data from a previous study, one of our co-authors (DvdH) found that the hippocampal tissue of female rat pups exposed to prenatal stress showed a 36% decrease in *Gabbr1* gene expression. In general, gene expression in these pups showed an involvement of GABAergic neurotransmission [45]. We hypothesize that the GABAergic network, in particular *GABBR1*, could play an important role in prenatal stress and that regulation via *GABBR1* DNA methylation may potentially influence HPA axis response.

Furthermore, we showed that fetal *GABBR1* DNA methylation at CpG_1.2 was associated with HPA axis reactivity in response to a routine vaccination at 4 months old. The observation that we find significant associations of *GABBR1* DNA methylation with stress response at 4 months but not at 2 months is likely due to the smaller sample size at 2 months of age, when only 20 out of 80 mother-child dyads participated. The lack of significant associations with infant cortisol day profiles at 12 months could be due to the fact that we could not observe an awakening response. Most likely, an increase of cortisol between awakening and 30 min could not be observed because infants were already lying awake for some time before the mothers could obtain the first cortisol sample of the day.

When comparing previously published EpiTYPER DNA methylation data of *NR3C1*-1F, *IGF2*, and *GNASXL* regions to the current HM450 data, we noticed that most analyzed CpG sites were not present on the HM450 chip. In the *IGF2*-DMR0 region, no probes were present. The *GNASXL* and *NR3C1*-1F regions were represented by one and five probes, respectively, which were not significant.

Some limitations to our current study should be acknowledged. First, the sample size of our genome-wide study is relatively small and therefore no CpG sites survived multiple testing corrections at the probe-by-probe (DMP) analysis level, which is often the case in similar studies where expected effect sizes are small [46, 47]. Instead, we focused on the validation of our findings by examining nearby CpG sites in a DMR analysis, additionally performing a validation study of our candidate gene region using EpiTYPER. Moreover, this current study was intended as a hypothesis-generating study in which we identified and validated *GABBR1* as a gene of interest, which was consistent with findings in prenatally stressed rodent brain tissue and associated with infant phenotype, i.e., HPA axis response. Second, we observe a cortisol peak at 15 min post-vaccination, as found by Lewis and Thomas [18]. However, as we have measured cortisol at 15, 30, 45, and 60 min following vaccination, we are presumably not measuring the highest cortisol response peak which is at 20–25 min post-stressor, by consensus [48, 49]. Yet, it is reasonable to assume that the AUC and slope values do reflect the inter-individual variability in cortisol response. Also, the fact that we could not observe an awakening response at 12 months is a major limitation for this part of the study. A delay in sampling at awakening and diurnal sampling during only 1 day likely limit the validity of this measurement. Third, we examine DNA methylation patterns in umbilical cord blood samples in response to pregnancy anxiety. There has been much debate on whether peripheral blood samples can be used as a surrogate tissue to study brain-related phenotypes. Although DNA methylation indeed shows tissue-specific differences [50], several published studies report valuable DNA methylation differences associated with disease found in peripheral tissues [51–53]. Moreover, data from the online Blood Brain DNA Methylation Comparison Tool [54] suggested that DNA methylation of the HM450 probes in the identified *GABBR1* DMR was correlated between peripheral blood and prefrontal cortex samples (most significant probe: cg05812266, *R* = 0.425, *p* = 0.00012). Furthermore, when analyzing genome-wide DNA methylation data from peripheral blood samples, it is important to take blood cell type composition into account, as these can vary between individuals and may confound associations of exposure [55]. A number of recent methodological studies question the validity of applying the Houseman algorithm [23] to cord blood samples for blood cell type correction [56–58]. However, the method has been extensively used in genome-wide DNA methylation studies using DNA from umbilical cord blood [59–66]. The Houseman algorithm estimations remain an approximate calculation, although they are a good alternative to correct for the potential influence of cell type composition in the absence of actual cell type measurements. The results presented in the current study should be interpreted with caution, taking these limitations into account.

## Conclusions

In conclusion, we showed that pregnancy anxiety is associated with fetal DNA methylation changes, identifying *GABBR1* as one of the top candidate genes associated with pregnancy anxiety in male newborns. We further provide evidence for a potential link of *GABBR1* DNA methylation with infant HPA axis reactivity to a stressor. Future longitudinal studies performed on larger cohorts are needed to verify and further explore these findings.

## Additional files

Additional file 1: Figure S1. Schematic overview of the study design from data processing to validation of our main findings. **Figure S2.** Overview of the *GABBR1* gene based on UCSC genome browser. **Figure S3.** Salivary cortisol measurements of infants at 2, 4 and 12 months old. (DOCX 1379 kb)

Additional file 2: *GABBR1* CpG sites analyzed by EpiTYPER and corresponding HM450 CpG probe identifiers. (XLSX 9 kb)

Additional file 3: Top 50 *DMRcate* results. (XLSX 17 kb)

Additional file 4: ErmineJ Gene Ontology (GO) pathways analysis results based on the top 500 annotated differentially annotated regions. (XLSX 14 kb)

Additional file 5: Top 100 ranked CpG probes associated with prenatal anxiety. (XLSX 20 kb)

Additional file 6: Overview of CpG probes in the *GABBR1* region identified by *DMRcate*. (XLSX 14 kb)

Additional file 7: Correlations between HM450 CpG probes and their respective CpG units measured by Sequenom EpiTYPER (*n* = 41). (XLSX 12 kb)

## Abbreviations

AUCi: Area under the curve with respect to increase
CAR: Cortisol awakening response
DMP: Differentially methylated CpG probe
DMR: Differentially methylated regions
EWAS: Epigenome-wide association study
FDR: False discovery rate
*GABBR1*: GABA-B receptor subunit 1 gene
*GNAS*: Guanine nucleotide binding protein, alpha stimulating
GO: Gene Ontology
HM450: HumanMethylation450 BeadChip
*HOXC4*: Homeobox C4
HPA: Hypothalamic-pituitary-adrenal
*IGF2*: Insulin-like growth factor 2
LINE: Long interspersed nuclear element
*NR3C*1: Glucocorticoid receptor gene
PELS: Prenatal Early Life Stress
*PPT2*: Palmitoyl-protein thioesterase 2
PRAQ: Pregnancy-related anxiety questionnaire
SNP: Single nucleotide polymorphism
