# Supplementary material for: Newborn genome-wide DNA methylation in association with pregnancy anxiety reveals a potential role for GABBR1
Source: Clin Epigenetics. 2017 Oct 3;9:107. doi: 10.1186/s13148-017-0408-5 (PMC5627482; doi:10.1186/s13148-017-0408-5)

Supplementary Figure 1: Schematic overview of the study design from data processing to validation of our main findings.

Following data preprocessing and quality control (QC), we analyzed HumanMethylation450 (HM450k) data for differentially methylated regions (DMR) and probes (DMP). From the top 10 identified DMRs, the GABA-B receptor (*GABBR1*) DMR was selected and further analyzed using a second DNA methylation quantification technique, Sequenom EpiTYPER in a larger cohort (n=80). EpiTYPER data were then used to examine the association between newborn *GABBR1* methylation and child cortisol levels in response to a stressor at two and four months of age, and in the cortisol awakening response at 12 months old.


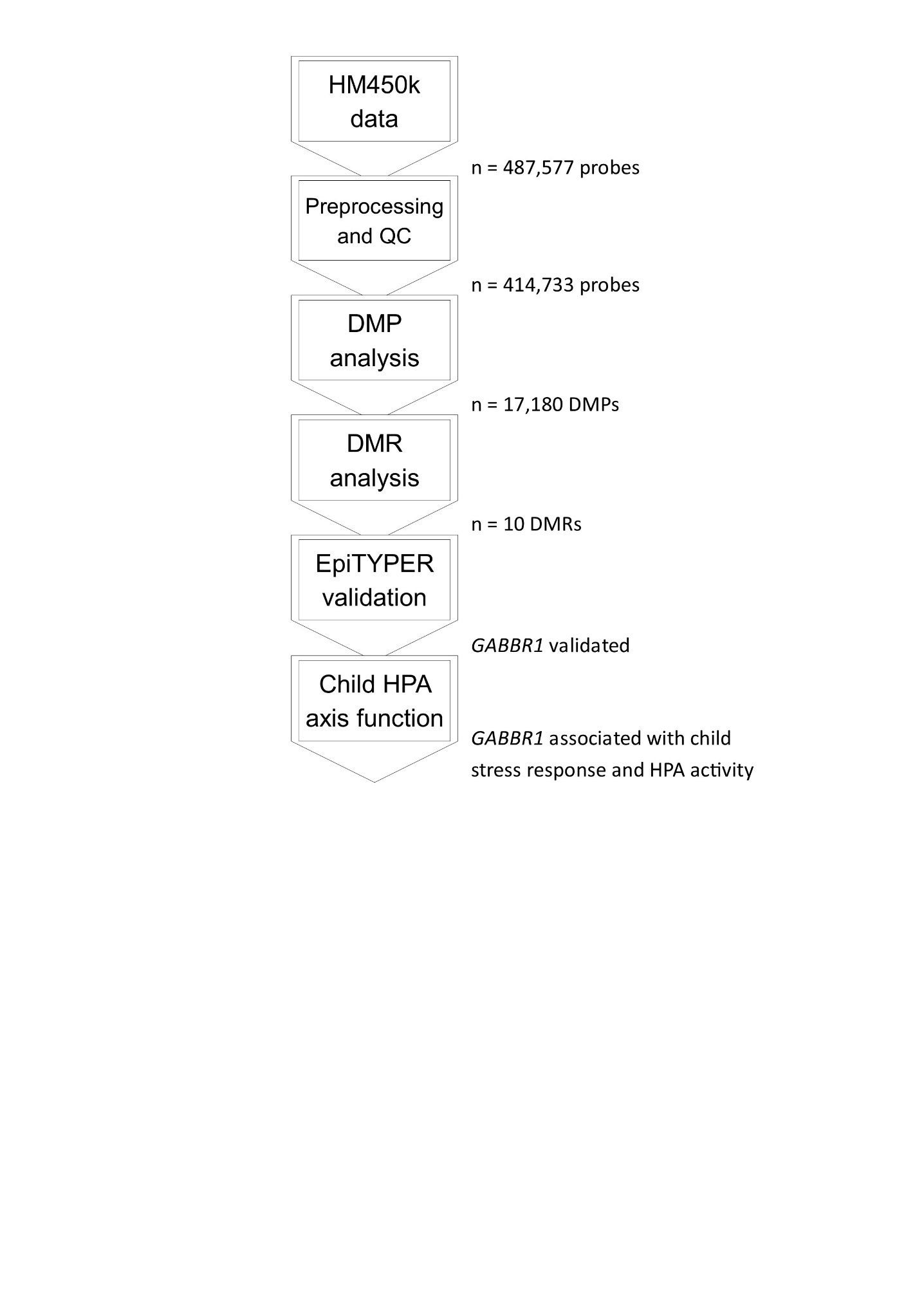


Supplementary Figure 2: Overview of the *GABBR1* gene, situating the *GABBR1* DMR amplicon for EpiTYPER analysis, based on UCSC genome browser (<http://genome.ucsc.edu/>). Includes the genetic sequence of the amplicon with CpG sites indicated with the exon part of the sequence underlined.


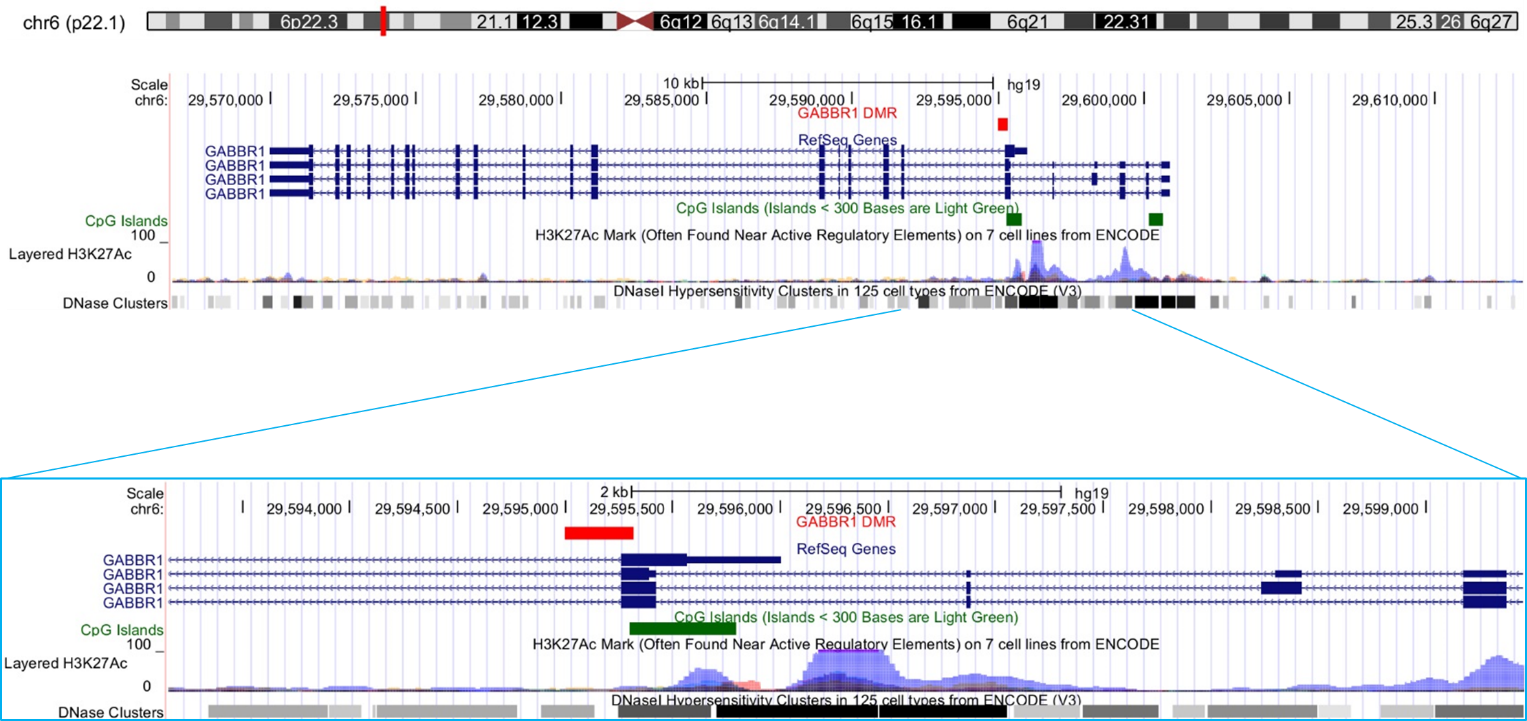


GGCCAGGGGGCCAGGCCTGCCAGCCCG^1^CG^2^GTGGAGATGGCG^3^CTGGAGGACG^4^TGAATAGCCG^5^CAGGGACATCCTGCCG^6^GACTATGAGCTCAAGCTCATCCACCACG^7^ACAGCAAGGTAGCCCTGGACATGGGGGTGGGTGGGAGGTGGGGGCTTGCG^8^GGGCAGGGGGCCAGCCAGCTGCACG^9^CG^10^CCCCCATCTGTCTGAGTCG^11^TCTCTGGGATTGCG^12^AGGCAGACCCCTCCCTTGTGTGACTGGCAGGAGATGGGCTGGGGGTGCAGGAGCTTGGGGAGAGTCG^13^CAGGGGCTGGAGGTCCAAGATGAGGGTCTAGGGGCTCAAGATGGTTAAGCATGCTGCAAGGCAGACCCTTCTGCCCCG^14^CTGCG^15^GGAGTCTCG^16^CAGAAGTGTCG^17^GGGTTTGGAGAAACTGGTGGTGGATTTAAGGTATTAGGAGACACTGATCCTCTGAGGGAGT

Supplementary Figure 3: Salivary cortisol measurements of infants a) at 2 and 4 months old, at baseline (T0), 15 minutes, 30 minutes, 45 minutes and 60 minutes following vaccination; b) at 12 months old, at awakening (T0), 30 minutes, 4 hours and 12 hours following T0. Error bars indicate 95% confidence intervals. C) The corresponding table shows descriptives for the data and indicates whether variables were non-normally distributed and consequently Log transformed for subsequent analyses. AUCi: area under the curve with respect to increase; CAR: cortisol awakening response (value at 30 minutes minus T0).


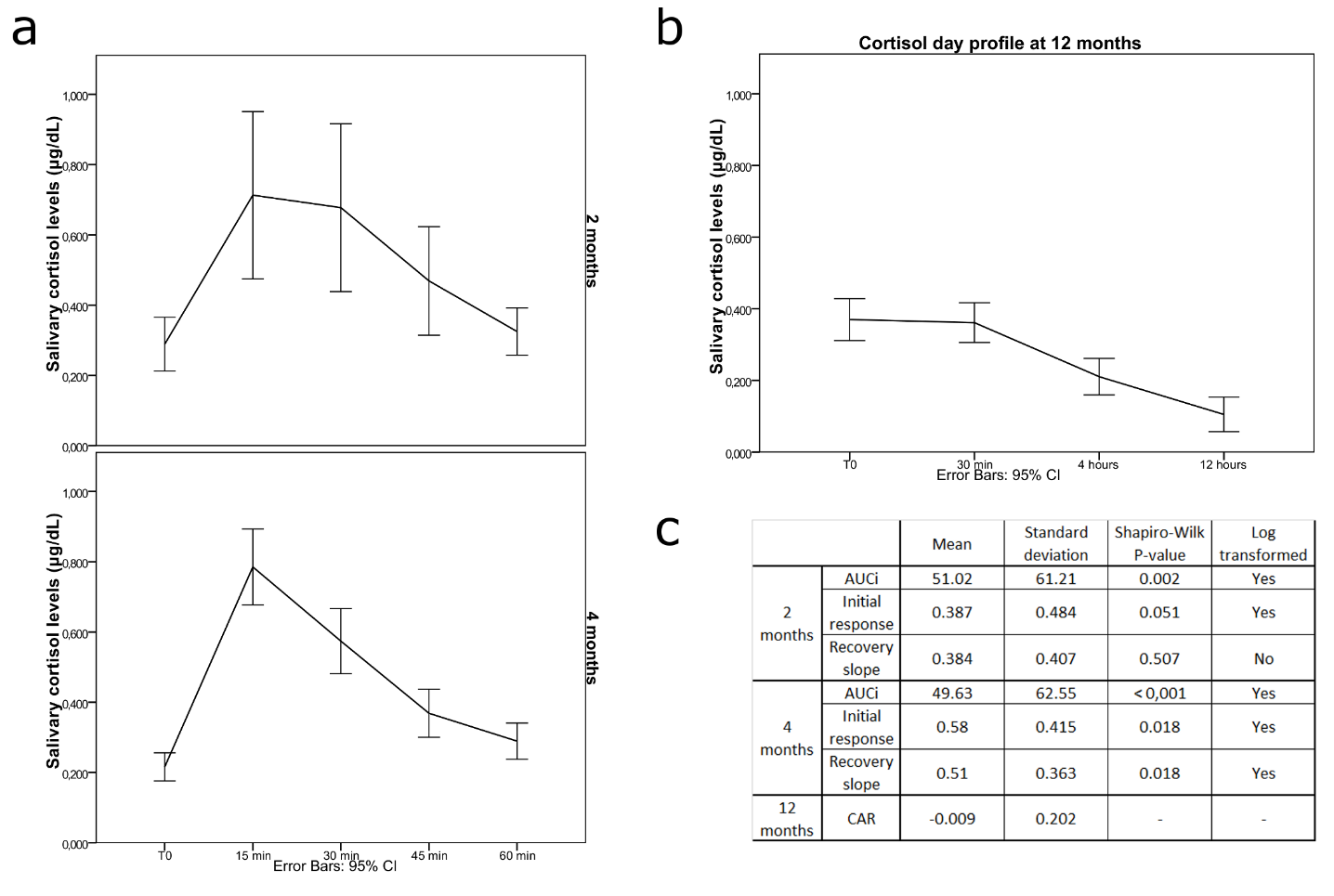

Supplement: Supplementary file 1 — Schematic overview of the study design from data processing to validation of our main findings. Figure S2. Overview of the GABBR1 gene based on UCSC genome browser. Figure S3. Salivary cortisol measurements of infants at 2, 4 and 12 months old. (DOCX 1379 kb) [file 13148_2017_408_MOESM1_ESM.docx]
